# Left atrial volume measurement with automated border detection by 3-dimensional echocardiography: comparison with magnetic resonance imaging

**DOI:** 10.1186/1476-7120-7-16

**Published:** 2009-03-31

**Authors:** Ramin Artang, Raymond Q Migrino, Leanne Harmann, Mark Bowers, Timothy D Woods

**Affiliations:** 1 Division of Cardiovascular Medicine, Medical College of Wisconsin, Milwaukee, Wisconsin, USA; 2 Department of Radiology, Medical College of Wisconsin, Milwaukee, Wisconsin, USA

## Abstract

**Objective:**

Left atrial size is an important marker for adverse cardiovascular events. There is general consensus that left atrial volume index (LAVI) is the best measurement of size. The current LAVI measurement techniques are laborious. Semi-automated measurement with a 3-dimensional echocardiography (3DE) system may be a practical clinical alternative to measure LAVI, but it has not been adequately evaluated against Magnetic Resonance Imaging (MRI) gold standard. The aim of this study was to compare the accuracy of a commercially available 3D algorithm for measurement of LAVI against LAVI obtained from MRI and Area Length Method (ALM).

**Design:**

In 27 consecutive subjects referred for cardiac MRI (age 54 ± 13 years, 63% male), LAVI was measured using 3 imaging modalities: 3DE, ALM, MRI and the results were correlated. ALM was measured using standard American Society of Echocardiography guidelines. The time required to measure LAVI by 3DE and ALM were compared.

**Results:**

There was a significant correlation in systolic and diastolic LA volumes and left atrial ejection fraction between 3DE and MRI (r = 0.86 for systole, r = 0.76 for diastole, r = 0.88 for ejection fraction, *P *< 0.0001 for all). There was also significant correlation of diastolic volumes between 3DE and ALM (r = 0.77, *P *< 0.0001). The time to obtain LAVI was shorter using 3DE versus ALM (56 ± 8 vs 135 ± 55 seconds, *P *< 0.0001).

**Conclusion:**

Three-dimensional echocardiography with semiautomatic border detection is a practical alternative for obtaining the left atrial volume in a time-efficient manner compared to the current standard.

## Background

Left atrial (LA) size is an independent marker of adverse clinical outcomes in conditions such as atrial fibrillation, myocardial infarction and heart failure [1-6]. Although measurement of a linear anterior-posterior LA dimension by M-mode or two-dimensional (2D) imaging has been the standard indicator of size for the past few decades, there is increasing recognition that enlargement occurs eccentrically which reduces the sensitivity of this measurement. Measurement of LA volume indexed to body surface area (BSA) is a more sensitive indicator of LA size. The Area Length Method (ALM) is currently recommended by the American Society of Echocardiography (ASE) as the preferred 2D method of estimation [7]. Previous studies using off-line 3-dimensional (3D) reconstruction of the left atrium that were obtained by time-sequenced 2 dimensional images correlated well to the gold standard of magnetic resonance imaging (MRI). But these methods were clinically impractical due to need for specialized software and prolonged time gap from image acquisition to volume calculation [8,9]. With the recent development of 3D matrix array transducers that can rapidly acquire real-time 3D images and the simultaneous development of semi-automated computer algorithms that can measure left ventricular volume from these images without using geometric assumptions, we hypothesized that this algorithm could be applied to estimate left atrial volume index in a time-efficient manner. The aim of this study was to apply the commercially available 3D semi-automated volume algorithm to the measurement of left atrial volume index, and validate its accuracy against the MRI gold standard as well as the ASE recommended 2D based area-length method. We further sought to compare the inter- and intraobserver variability, as well as the measurement time between 3DE and standard 2D ALM method.

## Methods

### Patient Selection

This study was approved by the Medical College of Wisconsin's Institutional Review Board. From April to December 2006, subjects who were referred for cardiac MRI for any indication were asked to participate in the study consecutively. Other inclusion criteria were age 18 years or older and willingness to provide informed consent. Exclusion criteria were presence of prosthetic valves, supraventricular or ventricular tachyarrhythmias at the time of image acquisition, known congenital heart disease and inability to complete cardiac MRI study due to claustrophobia. All consenting subjects were included and no subjects were excluded due to suboptimal image quality.

### Echocardiography

**Figure 1:**
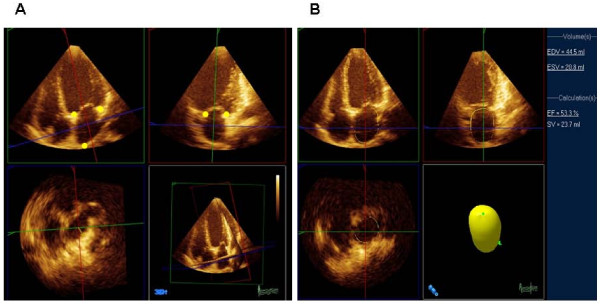
**Semi automatic left atrial border tracing by marking (●) at 4 mitral annular points (lateral, septal, inferior, anterior) and an atrial superior dome point opposite the annulus (A)**. The automatic border tracing is then shown by the software. The left atrial end diastolic volume (EDV), end systolic volume (ESV) and ejection fraction (EF) are calculated automatically by the software and displayed on the right side of the screen (B). SV = stroke volume.

Patients were positioned in the left lateral decubitus position. The echocardiogram was either obtained immediately following MRI or within 48 hours before or after the MRI. All measurements were performed off-line using Xcelera, QLAB and 3DQ-Advanced software (Phillips Medical Systems, Andover, MA). The real-time 3D images were acquired using an X3-1 matrix transducer on Phillips IE33 (Phillips Medical Systems, Andover, MA). Apical full-volume images were acquired over 4 cycles. The image was aligned in order to obtain the optimal border delineation of the left atrium in the far field. The 3D longitudinal axis was aligned parallel to the left atrium's axis. The 3D transverse axis was placed at the level of the left atrium where it crossed the 3D longitudinal axis approximately at the left atrium's geometric center point (Fig 1A). Maximum LA volume was measured in the frame just before mitral valve opening. The minimum LA volume was measured in the frame just after the P wave at mitral valve closure [10]. In this manuscript the maximum and minimum LA volumes are referred as LA diastolic and systolic volumes. Semi automatic left atrial border tracing was performed in LA systole and diastole by marking 4 mitral annular points (lateral, septal, inferior, anterior) and an atrial superior dome point opposite the annulus (Fig. 1A). The LA diastolic and systolic volumes were automatically calculated by the software and indexed manually to BSA. Furthermore the LA ejection fraction was automatically calculated by the software using the formula: [(LA diastolic volume - LA systolic volume)/LA diastolic volume] × 100, (Fig. 1B). The 2D images were acquired either on Phillips IE33 or General Electric Vivid 7 (General Electric Healthcare, Milwaukee, Wisconsin). The 2D images were obtained in standard apical 4 and 2 chamber views. The LA volume index was calculated using biplane ALM as recommended by the ASE Guidelines [7]. The volume index was calculated using a custom calculator software by entering the values of A1, A2, L and BSA. The formula applied was: A1 × A2 × 0.85/L × BSA, where the A1 is the area in apical 4 chamber view, A2 is the area in the apical 2 chamber view and L is the shorter length of the LA. The raw data was downloaded into the Xcelera digital reporting system for final analysis. The time to download the raw data was independent of whether the data included 2D or 3D images.

The time required to measure the LA volume by ALM and 3D techniques were compared. The 3D measurement time period included the interval from launching the 3D application, aligning the image for LA volume calculation in atrial diastole and then marking the 5 points until the border tracing algorithm was completed and then indexed manually to body surface area. Timing of the ALM began with area and length measurements, and included the time to enter the measured values and body surface area into the custom calculator. The measurements were performed independently by authors (RA and LH) who were blinded to MRI results.

### Magnetic resonance imaging

**Figure 2:**
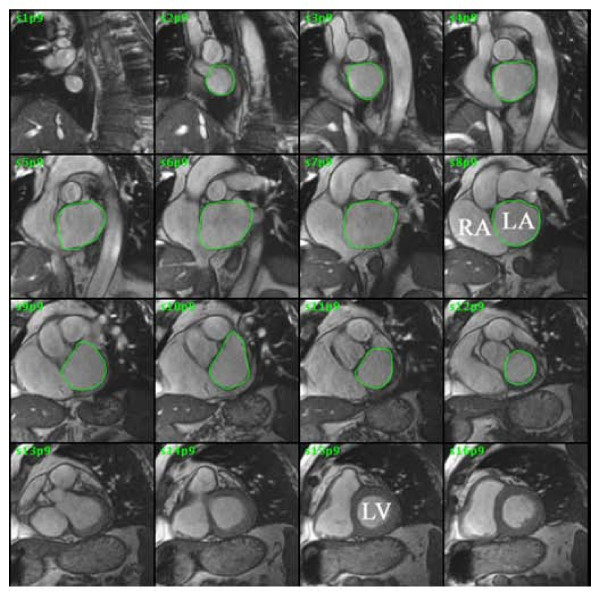
**MRI of left atrium**. Steady state free precession cine gradient echo was performed on sequential slices of the left atrium. The left atrial border was traced at end-left ventricular systole. The summation of the areas multiplied by slice thickness is the left atrial volume. LA = left atrium, RA = right atrium, LV = left ventricle.

Left atrial volume index was measured using cardiac gated steady state free precession cine pulse sequence of contiguous short axis slices (field of view 34–42 cm, slice thickness 6–8 mm, interslice gap 0–3 mm, flip angle 45 degrees, retrospective gating, temporal resolution 24–54 ms). The left atrial border was manually traced (Figure 2). The anterior border was at the mitral annular plane and the posterior border was the ostia of the pulmonary veins. The timing of the maximum and minimum LA volume measurement and calculation of LA ejection fraction was identical to that described under Echocardiography. Left atrial volumes were calculated using summation of area × (slice thickness + interslice gap) for each slice (Simpson's method) (MASS software, Leesburg, VA) and indexed to body surface area. The MRI was measured by authors (RQM and MB) who were blinded to echocardiography results.

### Statistical analyses

Data are expressed as means ± standard deviation unless otherwise indicated. The relation between any 2 methods was determined using Pearson's correlation. The Bland-Altman method was used to measure the limits of agreement between 2 methods. The time required to measure the LA volume by ALM and 3DE was compared using the Mann-Whitney test. Intraobserver and interobserver variability was determined for 3DE, MRI and ALM using coefficient of variability, calculated as the mean of absolute differences between 2 measurements divided by the average of the 2 measurements times 100 expressed as a percentage. Using previously published MRI values for left atrial volume in normal subjects [11], each participant was categorized as having either enlarged (>1 standard deviation from published mean) or normal left atrial size (≤ 1 standard deviation from published mean) by MRI. A receiver operating characteristic curve was calculated to determine the sensitivity and specificity of LAVI for both 3DE and ALM to detect left atrial enlargement. Two sided p-value was set at 0.05 for significance. Statistical analyses were performed using SPSS 13.0 (SPSS Inc., Chicago IL) and Sigmastat 3.5 (Systat Software Inc., Richmond CA).

## Results

Thirty three subjects were included in the study. Six subjects did not complete the MRI due to claustrophobia or ventricular arrhythmia. The final analysis was performed on 27 subjects who completed both MRI and echocardiography image acquisition. The median time window between MRI and echocardiography was 42 hours (total range 1.3 – 499 hours). Clinical characteristics are detailed in Table 1.

**Table 1:** Baseline characteristics of the included subjects

Number of subjects	27
Age (years) (mean ± standard deviation)	54 ± 13
Male (%)	17 (63)
Indication for MRI (%)	
Myocardial viability	9 (33)
Amyloidosis	3 (11)
Congestive heart failure	6 (22)
ARVD†	6 (22)
Coarctation of Aorta	1 (4)
Pulmonary artery stenosis	1 (4)
Patent ductus arteriosus	1 (4)

† Arrhythmogenic Right Ventricular Dysplasia

**Figure 3:**
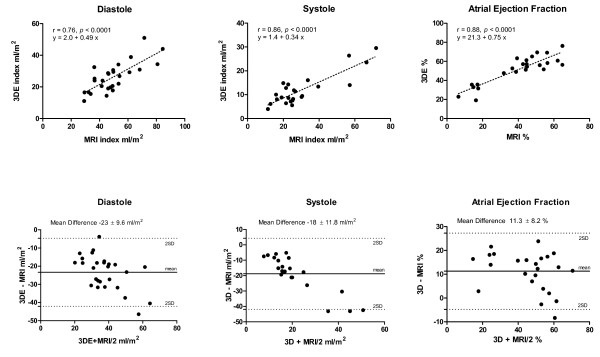
**The correlation analysis (top) and Bland-Altman analysis (bottom) of comparison between 3 dimensional echocardiography (3DE) and magnetic resonance imaging (MRI)**. SD = Standard Deviation.

**Figure 4:**
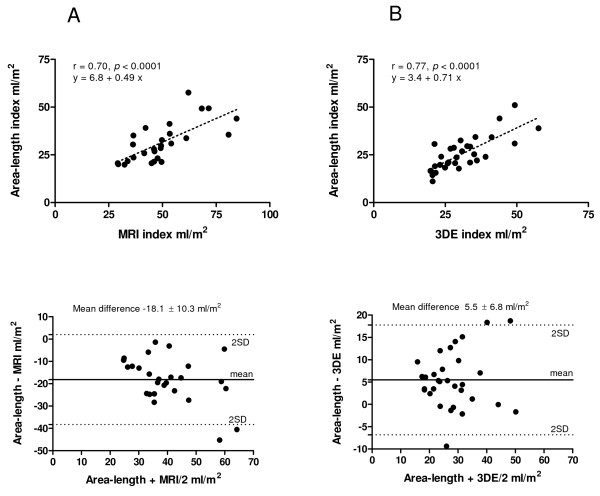
**The correlation analysis between diastolic volumes by area-length method (ALM) vs. magnetic resonance imaging (MRI) (A), and 3 dimensional echocardiography (3DE) vs. ALM (B)**.

There was a significant correlation between left atrial volumes obtained by 3DE and MRI (Fig 3). The correlation coefficients and mean differences (Δ) were r = 0.76, Δ -23.3 ± 9.6 ml/m^2 ^(*P *< 0.0001) for diastolic volumes, r = 0.86, Δ -18 ± 11.8 ml/m^2 ^(*P *< 0.0001) for systolic volumes, and r = 0.88, Δ 11.3 ± 8.2, (*P *< 0.0001) for left atrial ejection fraction. The diastolic volumes measured by ALM showed significant correlation with MRI as well (r = 0.70, *P *< 0.0001, Δ -18 ± 10) (Fig. 4A). There was a significant correlation between diastolic volumes measured by 3DE and ALM (r = 0.77, *P *< 0.0001, Δ 5 ± 6 ml/m^2^) (Fig. 4B). When the automated measurement of LA diastolic volumes by 3DE were manually corrected, the correlation with MRI was similar to the non-corrected measurements (r = 0.72, Δ -22 ± 10.1, *P *< 0.0001).

The time to calculate LA diastolic volume was shorter using 3DE versus ALM (56 ± 8 sec vs. 135 ± 55 sec, *P *< 0.0001). When the 3DE measurements were manually corrected, the time to calculate LAVI was 64 ± 15 sec (*P *< 0.0001 compared to ALM). The intra- and interobserver variability among the different methods is demonstrated in Table 2. The 3DE had comparable intra- and interobserver variability to MRI and more favorable variability as compared to ALM. The receiver operating characteristic curve analysis revealed significant sensitivity and specificity for atrial enlargement by both 3DE and ALM with area under the curve of 0.857 for 3DE (*P *= 0.006) and 0.893 for ALM (*P *= 0.002). Using likelihood ratio analysis (sensitivity/(1-specificity)), LAVI of 31 mL/m2 (for 3DE) and 33 mL/m2 (for ALM) had the highest sensitivity (86%) and specificity (80%) to detect left atrial enlargement based on MRI gold standard.

**Table 2:** The intra- and interobserver variability among the different methods in percent

**Method**	**Intraobserver **(%)	**Interobserver **(%)
Semiautomated 3D Technique	2	5.1
Magnetic Resonance Imaging	1.8	6.4
Area Length Method	9.9	12.4

## Discussion

In this study we compared for the first time the LA volume measured by a commercially available 3-dimensional echocardiography system that utilizes semiautomated border detection, to an MRI gold standard and demonstrated good correlation. This technique has furthermore proven to have favorable intraobserver and interobserver variability compared to MRI and ALM.

Measurement of LA volume by 3D echocardiography in adult patients has been attempted in the past by Keller and Rodevand in 2 separate studies [8,9]. The correlation with an MRI gold standard was excellent. The image acquisition, however, was obtained on a standard 2D transducer, with 3D reconstruction occurring off-line by specialized software. The off-line image processing took 6–10 minutes for LA volume measurement, making this a clinically impractical approach. Jenkins and co-workers applied real-time 3D echocardiography for measurement of the LA volume in 106 subjects [12]. The study compared different 2D methods of volume estimation to the 3D technique, but did not include an MRI gold standard. Furthermore, the study by Jenkins, et al [12] used a different software algorithm (4D analysis, Tomtec Gmbh, Unterschlessheim, Germany) for LA volume estimation, requiring almost 8 times longer processing time (430 ± 15 s vs 56 ± 8 s in the present study). Similar to the present study, they found significant correlation between the volumes obtained by the ALM and the 3D technique.

The accuracy of MRI measurement of left atrial volume has been validated against water displacement of cadaveric atrial casts by Järvinen and coworkers [13]. Our findings show that echocardiography systematically underestimates the LA volumes as compared to MRI. This phenomenon has been described from previous comparisons of echocardiography versus MRI and gated cardiac computer tomography for assessment of left atrial and ventricular volumes [9,14,15]. A likely explanation is the difference in spatial image resolution between imaging techniques. In both 2D and 3D echocardiography, the apical window places the left atrium at the far field of the ultrasound beam, resulting in loss of lateral image resolution. In contrast to MRI, planimetry of 2D and 3D ultrasound images may not distinguish the volumes within the intratrabecular areas [14]. As illustrated in figures 1 and 2, the greater image resolution of MRI permits more accurate border detection of the left atrium as compared to 3DE that might explain an average of 15–20 ml difference in volume observed between modalities in the present study and in the literature. The underestimation of atrial diastolic and systolic volumes by 3DE resulted in mild overestimation of atrial ejection fraction compared to MRI (Fig. 3). Left atrial ejection fraction has been associated with left ventricular systolic and diastolic function [16]. Routine use of left atrial ejection fraction is currently not part of the daily clinical practice likely due to the additional time required to obtain this parameter. With 3DE, atrial volume data at multiple time points per cardiac cycle and LA function are automatically calculated by the software in a time interval that is comparable or shorter than conventional ALM. Larger studies are however required to correlate the atrial ejection fraction assessed by 3DE to systolic and diastolic dysfunction.

In this study we demonstrated that time to obtain LAVI was significantly shorter using 3DE even with manual correction as compared to ALM measurements when measured off-line. Measurements using both techniques were performed off-line to replicate best clinical-practice guidelines. Although many modern ultrasound systems permit on-line ALM calculation of LAVI, a critical shortcoming is the inability to adjust sonographers' left atrial area tracings by the interpreting physician off-line. In our multi-vendor clinical laboratory, we have overcome this limitation by performing all measurements off-line on images already transferred to the digital reporting system.

Our findings suggest that in laboratories equipped with 3D matrix-array transducers and an off-line quantification application, the 3DE is the most time-efficient method of LA volume quantification. This finding, along with good MRI correlation and reproducibility, implies this may be the preferred method of LAVI measurement except in cases when poor image quality prevents automated border detection. Given the prognostic implication of LA size, the ease of 3D derived volume and ejection fraction calculation will allow more routine acquisition of these parameters. In a busy echocardiography imaging laboratory the time saved by the sonographers and readers to calculate such parameters might have significant impact on the efficiency of the lab as well as better patient care.

## Limitations

This study was limited by a relatively small sample size. Although we did not have normal subjects, the use of subjects with suspected heart disease makes this study applicable to clinical practice. The study population was biased towards subjects who are undergoing MRI. All subjects had adequate echocardiography acoustic windows and no one was excluded due to poor echocardiographic image quality. Therefore, the study is not biased in terms of echocardiographic imaging quality limitation. The group studied more closely reflects the referral of a tertiary institution with a higher proportion of patients with advanced cardiac disease. The findings should be validated in a larger and more general population reflecting the continuum of left atrial size. In this study we used commercially available software that was originally designed for assessment of left ventricular volume and function and applied it to the measurement of LA volume which explains the ellipsoid shape of the left atrium illustrated in Figure 1B. In spite of these limitations, the correlation with MRI measurements was strong. These are the first steps in the application of this method for left atrial volume measurement. The data should motivate the development of software specifically designed for left atrial geometry that might improve the accuracy of this method.

## Conclusion

Three dimensional echocardiography with semiautomatic border detection is a practical alternative for obtaining the left atrial volume in a time-efficient manner compared to the current ASE standard and has good correlation with MRI measurements.

## Abbreviations

3DE: 3 dimensional echocardiography; ALM: area length method; ASE: American Society of Echocardiography; LAVI: Left atrial volume index; MRI: magnetic resonance imaging.

## Competing interests

The authors declare that they have no competing interests.

## Authors' contributions

RA was responsible for the conceptualizing and the design of the study, echocardiographic measurements, the statistical analysis and preparation and submission of the manuscript. RQM was responsible for MRI image acquisitions, measurement and had a substantial contribution in finalizing of the manuscript and the statistical analysis. LH was responsible for the coordination of the study, echocardiographic image acquisition and measurement and reviewed the manuscript for relevant intellectual content, MB was responsible for MRI measurements and reviewed the manuscript for relevant intellectual content, TDW was the principal investigator, responsible for conceptualizing and the design of the study and had substantial contribution in finalizing the manuscript.
